# Genetic variations in genes of metabolic enzymes predict postoperational prognosis of patients with colorectal cancer

**DOI:** 10.1186/s12943-015-0442-x

**Published:** 2015-09-17

**Authors:** Guanglong Dong, Xianli He, Yibing Chen, Haiyan Cao, Jiaojiao Wang, Xiaonan Liu, Shukui Wang, Shaogui Wan, Jinliang Xing

**Affiliations:** Department of General Surgery, The General Hospital of PLA, Beijing, 100853 China; Department of General Surgery, Tangdu Hospital, Fourth Military Medical University, Xi’an, 710032 China; State Key Laboratory of Cancer Biology, Experimental Teaching Center of Basic Medicine, Fourth Military Medical University, 169 Changle West Road, Xi’an, Shaanxi 710032 China; Xijing Hospital of Digestive Disease, Fourth Military Medical University, Xi’an, 710032 China; Central Laboratory, Nanjing First Hospital, Nanjing Medical University, Nanjing, 210006 China; Institute of Pharmacy, Pharmaceutical College of Henan University, 85 Minglun Street, Kaifeng, Henan 475001 China

**Keywords:** Colorectal cancer, Prognosis, Single nucleotide polymorphism, Succinate dehydrogenase, Tricarboxylic acid cycle

## Abstract

**Background:**

Genetic alterations in tricarboxylic acid (TCA) cycle metabolic enzymes were recently linked to various cancers. However, the associations of single nucleotide polymorphisms (SNPs) in genes of these enzymes have not been well studied.

**Methods:**

We genotyped 16 SNPs from 7 genes encoding TCA cycle metabolic enzymes in 697 colorectal carcinoma (CRC) patients receiving surgical resection and analyzed their associations with clinical outcomes by multivariate Cox proportional hazard model. Then, the significant results were validated in another cohort of 256 CRC patients.

**Results:**

We identified 4 SNPs in 2 genes had significant associations with CRC death risk and 5 SNPs in 3 genes had significant associations with CRC recurrence risk. Similar significant results were confirmed for rs4131826 in *SDHC* gene, rs544184 in *SDHD* gene and rs12071124 in *FH* gene in a validation cohort. Further analysis indicated that unfavorable genotypes exhibited a significant cumulative effect on overall and recurrence-free survival in a dose-dependent manner. Moreover, survival tree analysis indicated that SNP rs4131826 in *SDHC* gene and SNP rs12071124 in *FH* gene were the primary factors contributing to the different overall survival time and recurrence-free survival time of CRC patients, respectively. Immunohistochemical analysis further validated the effect of rs4131826 and rs544184 on expression of SDHC and SDHD in tissue samples.

**Conclusions:**

Our study suggests that SNPs in TCA cycle metabolic enzymes might be significantly associated with clinical outcomes in Chinese population diagnosed with CRC. Further functional and validated studies are warranted to expend our results to clinical utility.

**Electronic supplementary material:**

The online version of this article (doi:10.1186/s12943-015-0442-x) contains supplementary material, which is available to authorized users.

## Introduction

Colorectal cancer (CRC) is the third most common malignancy leading to a major cause of cancer mortality and morbidity worldwide [1]. Recently, increased incidence rates of CRC have been observed in regions that previously had relative lower CRC risk, especially in China [2]. Specific and sensitive prognostic biomarkers which are capable of identifying CRC patients with benefit from treatment will improve patients’ outcomes. To date, TNM staging system is the only widely accepted clinical prognostic factor [3, 4], although many biomarkers, such as microsatellite instability (MSI), chromosomal instability (CIN) and mutations of some tumor suppressor genes, have been reported to be associated with CRC patients’ outcomes. Novel biomarkers that can be adapted to CRC clinical utility are still urgently needed.

Altered metabolism is a hallmark of cancer, contributing to the initiation, growth, and maintenance of tumors [5]. Tricarboxylic acid (TCA) cycle which occurs in mitochondria is a well-known central metabolic pathway in the metabolism of sugars, lipids and amino acids. Main regulators of TCA cycle consist of three key metabolic enzymes, *i.e.* succinate dehydrogenase (SDH), fumarate hydratase (FH), and isocitrate dehydrogenase (IDH). Recently, more and more evidence has suggested that mutations of metabolic enzyme genes in TCA cycle are involved in the development of cancer [6–8]. It has been found that loss-of-function mutations in genes of SDH complex and FH lead to the accumulation of their substrates, *i.e.* succinate and fumarate, respectively, while gain-of-function mutations of *IDH* with neomorphic enzyme activity produces a novel metabolite, D-2-hydroxyglutarate (2-HG). All of these metabolites have recently been found to be associated with cancer development [9–14]. Although many studies are focused on the genetic mutations of TCA cycle core enzymes in cancers, there is no study so far to investigate the roles of SNPs in genes encoding these enzymes in CRC prognosis.

Single nucleotide polymorphism (SNP) is the most common genetic variation, and numerous previous studies have shown that SNPs may be promising surrogate biomarkers to predict therapeutic responses or prognosis of cancer patients [15]. However, the association between TCA cycle enzyme genes and the prognosis of CRC has never been investigated. In the present study, we sought to systemically evaluate the associations between functional SNPs in genes encoding TCA cycle core enzymes, including all subunits of *SDH*, *FH*, and *IDH* genes, and postoperational clinical outcomes in a hospital-based Chinese patient cohort diagnosed with CRC.

## Materials and methods

### Study population

The subjects in this study were enrolled between May 2006 and June 2012 from Xijing Hospital and Tangdu Hospital affiliated to the Fourth Military Medical University in Xi’an, China. The enrolled patients have to match the following criteria: 1) histologically confirmed with primary colorectal adenocarcinoma and no history of other cancers; 2) received curative surgical resection treatment, but without any preoperative anticancer treatment; 3) with complete clinical and follow-up data, as well as common epidemiological data. After excluding 16 patients who died within 1 month after surgical resection, a total of 697 CRC patients were included in this analysis. An additional validation cohort of 256 CRC patients was recruited from Nanjing First Hospital in Nanjing, China based on same enrollment criteria. Prior to surgical resection, 5 ml of peripheral blood sample was collected from each patient for DNA preparation. This study was approved by the Ethic Committees of FMMU and Nanjing First Hospital. Written informed consents were obtained from all patients.

### Demographic and clinical data

Demographic variables were collected by in-person interviewing using a standardized epidemiological questionnaire including gender, age, tumor position, differentiation, stage, and chemotherapy. Major clinical data were collected from medical records and consulting with the treating doctors, including variables of tumor position, TNM stage, tumor differentiation, and the adjuvant chemotherapy. The standard follow-up was updated at 6-month intervals through onsite interview, direct calling, or medical chart review by trained clinical specialists. The latest follow-up data in our analysis was obtained in January 2014 for both patient cohorts.

### SNP selection and genotyping

The candidate functional SNPs in TCA cycle metabolic enzymes were selected by web-based SNP selection tools (http://snpinfo.niehs.nih.gov/snpinfo/snpfunc.htm). The potential functional SNPs were selected based on the following criteria: 1) the SNPs had minor allele frequency ≥5 % in Han Chinese population (CHB) in the HapMap database; 2) the position of candidate SNP should be located in miRNA binding sites of 3’-UTR region, in transcription factor binding site of 5’-flanking region (2000 bp upstream from the transcription start site), or in mRNA splice site or exons. If there were multiple functional SNPs within the same haplotype block and the linkage coefficient r^2^ > 0.8, only one SNP was included. Finally, 18 potential functional SNPs in *SDH* genes (including subunits of *SDHA*, *SDHB*, *SDHC*, and *SDHD*), *FH* gene, and *IDH* genes (including subunits of *IDH1* and *IDH2*) were identified for genotyping with Sequenom iPLEX platform (Sequenom Inc., San Diego, CA, USA). Significant SNPs were also genotyped in validation cohort with Sequenom iPLEX platform. Strictly quality controls were implemented in each assay when genotyping and only those SNPs with call rate > 95 % were included for further analysis.

### Immunohistochemical staining of SDHC and SDHD

Formalin-fixed, paraffin-embedded CRC tissues from patients with different genotypes of SNPs rs4131826 and rs544184 were collected from Department of Pathology in Xijing Hopital. HE slides from these patients were viewed under a light microscope by a pathologist and 4-μm-thick tissue sections were cut from corresponding blocks containing representative tumor regions. Immunohistochemical staining of SDHC and SDHD was performed as previously described [16]. A rabbit anti-human SDHC antibody (1:200, Abcam) or rabbit anti-human SDHD antibody (1:200, Abcam) was used. The intensity and extent of immunostaining were evaluated for all samples under double-blinded conditions as previously described [16].

### Statistical analysis

In this study, the clinical outcomes of CRC patients include two major endpoints, overall survival (OS) and recurrence-free survival (RFS). Overall survival time was defined as the time from initial surgery to death from any cause. Recurrence-free survival time was defined as the time from initial surgery to local recurrence, distant recurrent metastasis. The associations between SNPs and CRC outcomes were estimated as hazard ratios (HRs) by Cox proportional hazards regression model with adjustment for gender, age, hospital site, tumor position, clinical stage, tumor differentiation and adjuvant chemotherapy. Three genetic models (dominant, recessive, and additive) were tested and the best fitting model (with smallest *P* value) was selected for further analysis. Kaplan-Meier survival curves and log-rank test were used to assess the differences in OS and RFS. We also calculated the false-positive report probability (FPRP) at prior probability levels of 0.001, 0.01, 0.1, and 0.25 to validate all statistically significant findings as previously described [17]. A FPRP value <0.1 was considered to be noteworthy. Cumulative effect was evaluated by the combination of unfavorable genotypes identified from the main effect analysis of individual SNP. The SPSS Statistics19.0 software (IBM) was used for all statistical analyses. Survival tree analyses were used to determine the higher-order gene-gene interactions, which were performed by the STREE program (http://masal.med.yale.edu/stree/) using recursive-partitioning to identify subgroups of individuals at higher risk.

## Results

### Characteristics of the study population

The distributions of 697 patients’ demographic and clinicopathologic characteristics by death or recurrence were summarized in Additional file 1: Table S1. During the median follow-up time of 35.7 months (range, 3.1-83.4 months), there were 205 patients (29.4 %) died and 240 patients (34.4 %) developed recurrence among CRC patients. Significant higher death and recurrence risks were observed in patients at late stage (stages III + IV) with HRs of 3.82(95 % CI 2.75-5.33, *P* < 0.001) and 3.13 (95 % CI 2.32-4.23, *P* < 0.001), respectively, when comparing to those at early stage (stages I + II). Expectably, patients with adjuvant chemotherapy exhibited significant protective effect on OS and RFS with HRs of 0.36 (95 % CI 0.25-0.52, *P* < 0.001) and 0.45 (95 % CI 0.32-0.63, *P* < 0.001), respectively, when comparing to those without adjuvant chemotherapy. There was no significantly different death or recurrence risk observed in patients with regard to gender, age, hospital site, tumor position, or tumor differentiation. Very similar demographic and clinicopathologic characteristics were observed in validation cohort of 256 CRC patients (Additional file 2: Table S2).

### The associations between individual SNPs and CRC clinical outcomes and gene expression

**Fig. 1:**
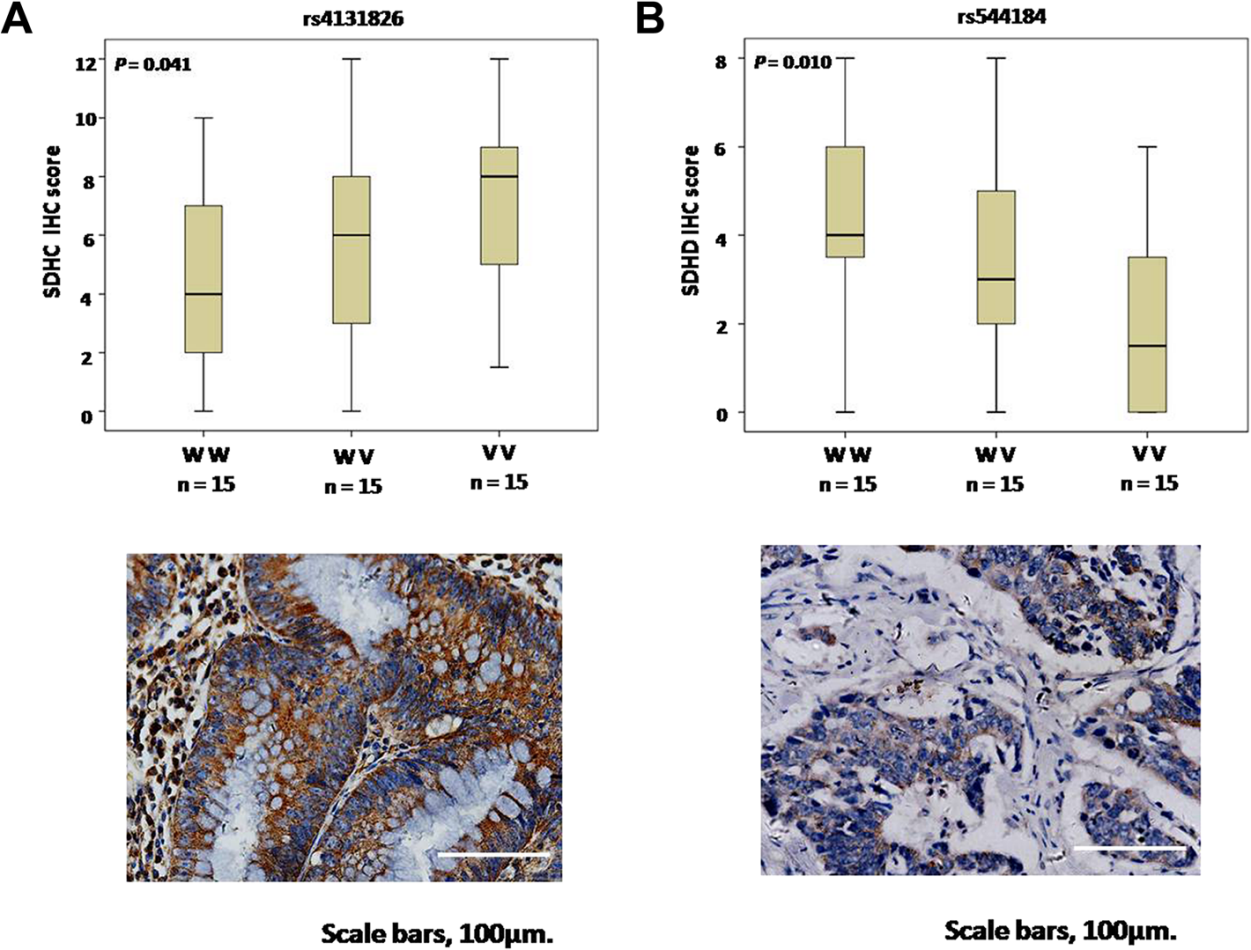
Immunohistochemical staining of SDHC (**a**) and SDHD (**b**) in tumor tissue samples from CRC patients with different genotypes of SNP rs4131826 or SNP rs544184

After excluding 2 SNPs with unqualified call rate (84.9 % for rs9708193 and 66.6 % for rs4932158), we eventually analyzed 16 SNPs in 7 genes encoding the TCA cycle core metabolic enzymes. We assessed the association of each individual SNP with CRC death or recurrence risk by multivariate Cox regression with adjustment for gender, age, hospital site, tumor position, clinical stage, tumor differentiation and adjuvant chemotherapy. As shown in Table 1, there were 2 SNP in *SDHC* gene and 2 SNPs in *SDHD* gene exhibited significant association with increased CRC death risk under additive and recessive models, respectively. And the most significant result was found in the association of rs4131826 of *SDHC* gene with OS (HR 0.61, 95 % CI 0.47-0.79, *P* < 0.001) under additive model. In addition, 2 SNPs in *SDHD* and *FH* genes exhibited borderline significant association with CRC death risk under additive model. In the RFS analysis, we found that 1 SNP (rs4131826) in *SDHC* gene, 3 SNPs (rs10789859, rs544184 and rs7121782) in *SDHD* gene, and 1 SNP (rs12071124) in *FH* gene had significant associations with CRC recurrence risk (Table 1). We calculated the false-positive report probability (FPRP) to validate all statistically significant findings. As shown in Additional file 3: Table S3, rs4131826 in *SDHC* gene and 3 SNPs in *SDHD* gene were considered to be noteworthy. Further analysis showed that 3 SNPs (rs10789859, rs544184 and rs7121782) in *SDHD* gene were located within the same haplotype block with strong linkage disequilibrium (LD = 0.947, 0.896 and 0.923 respectively). Therefore, we only select one (SNP rs544184) for further validation. As shown in Table 2 with a total of 4 validated SNPs, similar significant results were confirmed for rs4131826 in *SDHC* gene, rs544184 in *SDHD* gene and rs12071124 in *FH* gene, but not for rs12064957 in *SDHC* gene. We have further examined the expression of SDHC and SDHD in tissue samples from CRC patients with different genotypes of SNPs rs4131826 and rs544184 using immunohistochemical staining (IHC). As showed in Fig. 1, patients carrying VV or WV genotype of rs4131826 exhibited the significantly higher expression of SDHC when compared with those carrying WW genotype, and patients carrying WW or WV genotypes of rs544184 exhibited significantly higher expression of SDHD when compared with those carrying VV genotype.

**Table 1:** Association between SNPs in TCA cycle related genes and CRC outcomes in primary cohort

Gene	SNP	Call rate (%)	Function	OS			RFS		
				Best fitting model	HR (95 % CI)^a^	*P* value	Best fitting model	HR (95 % CI)^a^	*P* value
SDHA	rs13173911	99.0	TFBS	Additive	0.86(0.52-1.44)	0.573	Additive	0.88(0.55-1.40)	0.589
	rs2864963	98.4	TFBS	Dominant	0.92(0.69-1.21)	0.533	Dominant	0.92(0.71-1.19)	0.522
SDHB	rs3754509	98.0	TFBS	Recessive	0.96(0.66-1.40)	0.823	Recessive	0.88(0.62-1.27)	0.504
SDHC	rs12064957	98.9	TFBS	Additive	**1.36(1.06-1.74)**	**0.015**	Additive	1.21(0.95-1.54)	0.117
	rs3935401	98.7	miRNA	Recessive	1.25(0.68-2.31)	0.476	Dominant	0.91(0.70-1.19)	0.484
	rs4131826	96.1	TFBS	Additive	**0.61(0.47-0.79)**	**1.8x10** ^**-4**^	Additive	**0.73(0.58-0.91)**	**0.005**
SDHD	rs10789859	98.3	TFBS	Additive	1.19(0.97-1.45)	0.088	Additive	**1.29(1.08-1.55)**	**0.006**
	rs544184	96.8	TFBS	Recessive	**1.52(1.05-2.19)**	**0.026**	Additive	**1.31(1.08-1.58)**	**0.005**
	rs7121782	98.3	TFBS	Recessive	**1.49(1.04-2.14)**	**0.031**	Additive	**1.29(1.07-1.55)**	**0.008**
IDH1	rs12478635	97.4	TFBS	Additive	1.04(0.85-1.28)	0.682	Dominant	1.02(0.76-1.38)	0.875
IDH2	rs11540478	99.0	Splicing	Additive	0.91(0.69-1.19)	0.490	Additive	0.96(0.75-1.23)	0.731
	rs11632348	98.3	TFBS	Recessive	0.67(0.36-1.24)	0.203	Recessive	0.69(0.39-1.24)	0.218
	rs4283211	98.6	TFBS	Dominant	0.88(0.66-1.17)	0.387	Additive	0.94(0.75-1.18)	0.593
FH	rs12071124	97.4	TFBS	Additive	0.83(0.67-1.03)	0.086	Recessive	**0.62(0.43-0.90)**	**0.013**
	rs1414493	97.8	TFBS	Recessive	1.08(0.73-1.58)	0.709	Dominant	1.14(0.86-1.53)	0.359
	rs7530270	98.7	TFBS	Recessive	0.85(0.60-1.21)	0.361	Recessive	0.76(0.54-1.05)	0.100

Abbreviations: *CI* confidence interval, *HR* hazard ratio, *OS* overall survival, *RFS* recurrence free survival. Significant *p* values were in bold

^a^Adjusted for gender, age, hospital site, tumor position, clinical stage, differentiation and treatment after surgery

**Table 2:** Association between significant SNPs and 256 CRC patients’ outcome in validation cohort

Gene	SNP	Call rate (%)	Function	OS			RFS		
				Best Fitting Model	HR^a^ (95 % CI)	*P*-value	Best Fitting Model	HR^a^ (95 % CI)	*P*-value
SDHC	rs12064957	98.8	TFBS	Additive	1.16(0.79-1.70)	0.450	Additive	1.14(0.84-1.54)	0.406
	rs4131826	96.1	TFBS	Additive	**0.66(0.46-0.94)**	**0.022**	Additive	**0.73(0.55-0.97)**	**0.027**
SDHD	rs544184	97.3	TFBS	Recessive	**2.30(1.40-3.78)**	**0.001**	Recessive	**2.02(1.33-3.07)**	**0.001**
FH	rs12071124	96.5	TFBS	Additive	0.90(0.67-1.22)	0.511	Additive	**0.77(0.60-0.98)**	**0.033**

Abbreviations: *CI* confidence interval, *HR* hazard ratio, *OS* overall survival, *RFS* recurrence free survival. Significant *P* values were in bold

^a^Adjusted by gender, age,tumor position, clinical stage, differentiation and treatment after surgery where appropriate

### Cumulative effects of unfavorable genotypes on CRC clinical outcomes

To assess the cumulative effect of combined SNPs on CRC clinical outcomes, we grouped the patients by the number of unfavorable genotypes of SNPs with significant or borderline significant association under dominant model, and evaluated their associations with CRC death or recurrence risk by multivariate Cox proportional hazard model. As shown in Table 3, significant increased death risk were observed in patient groups with 2, 3, and 4 unfavorable genotypes, with HRs of 1.93 (95 % CI 1.18-3.18), 2.14 (95 % CI 1.28-3.58), and 3.17 (95 % CI 1.79-5.61), respectively, when comparing to group with no more than 1 unfavorable genotype. The cumulative death risk of CRC patients conferred by combined unfavorable genotypes exhibited a significant dose-dependent manner with *P* for trend < 0.001. Similarly, the significant increased recurrence risks were observed in patient groups with 2, 3, and 4 unfavorable genotypes, when comparing to those with zero or 1 unfavorable genotype. And also the increased combined recurrence risk exhibited a dose-dependent manner with *P* for trend = 0.001. As shown in Additional file 4: Table S4, very similar cumulative effect of unfavorable genotypes was also observed in validation cohort.

**Table 3:** Cumulative effect of unfavorable genotypes on CRC outcomes

Group(number of unfavorable genotypes^a^)	Death/Total	HR (95 % CI)^b^	*P* value	Recurrence/Total	HR^b^ (95 % CI)	*P* value
Group 1 (0-1)	20/138	Ref.		29/138	Ref.	
Group 2 (2)	78/246	**1.93(1.18-3.18)**	**0.009**	94/246	**1.81(1.19-2.76)**	**0.006**
Group 3 (3)	59/190	**2.14(1.28-3.58)**	**0.004**	68/190	**1.89(1.21-2.93)**	**0.005**
Group 3 (4)	31/69	**3.17(1.79-5.61)**	**7.30 × 10** ^**-5**^	31/69	**2.45(1.47-4.09)**	**0.001**
*P* for trend			**1.12 × 10** ^**-4**^			**0.001**

Abbreviations: *CI* confidence interval, *HR* hazard ratio, *Ref*. reference

^a^Unfavorable genotypes: SDHC rs12064957 (WV + VV) and rs4131826 WW, SDHD rs544184 (WV + VV), and FH rs12071124 (WW + WV)

^b^Adjusted for gender, age, hospital site, tumor position, clinical stage, differentiation and treatment after surgery

Significant *P* values (<0.05) were bolded

### Modulated effects of chemotherapy on CRC outcomes stratified by the number of unfavorable genotypes

**Fig. 2:**
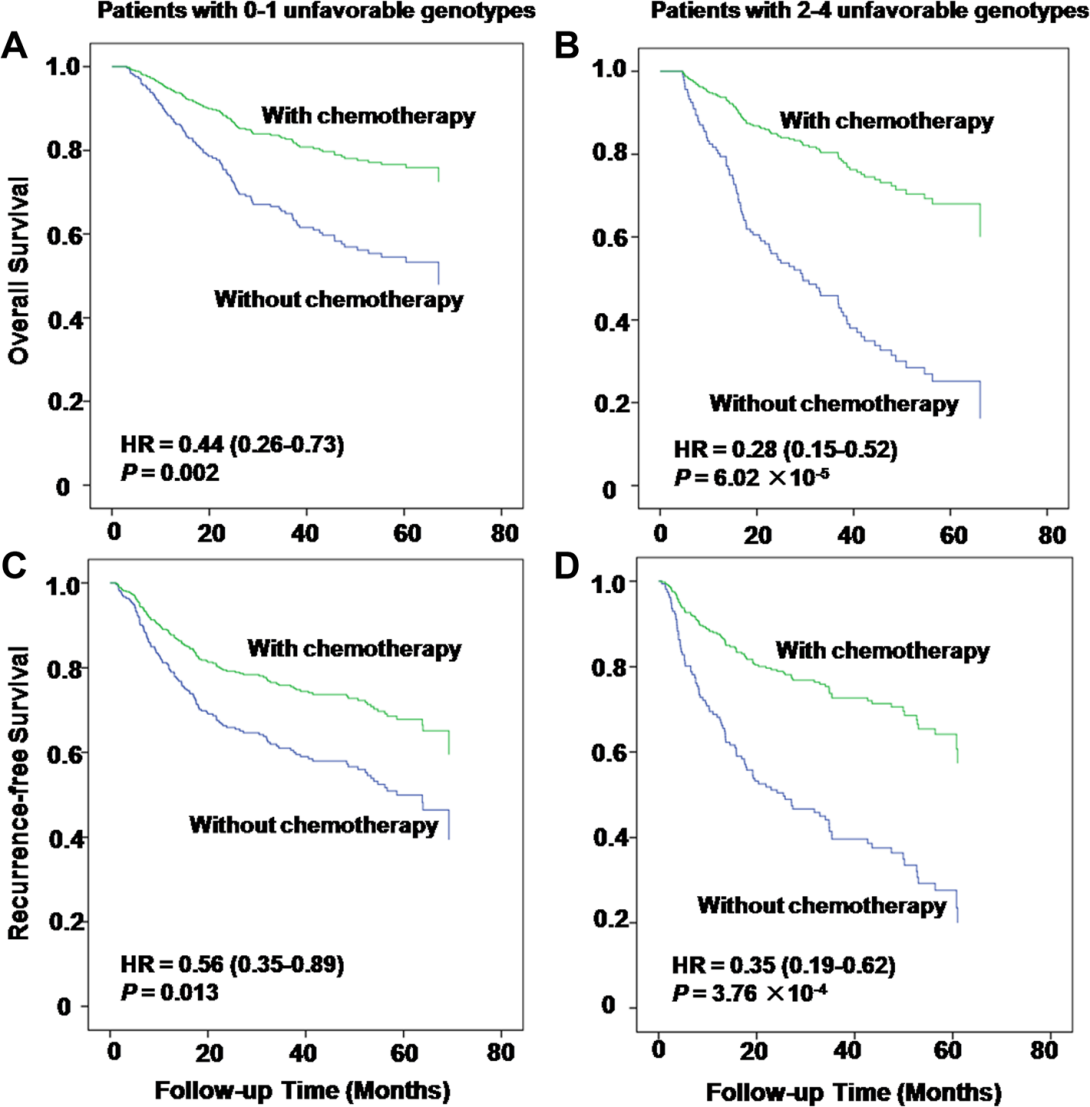
The protective effects of chemotherapy on CRC patients stratified by the number of unfavorable genotypes in the overall survival analyses (**a** and **b**) and in the recurrence-free survival analysis (**c** and **d**)

To evaluate whether the number of unfavorable genotypes could modify the protective effects of postsurgical adjuvant chemotherapy on CRC patients’ clinical outcomes, Kaplan-Meier curves analysis and log-rank test were performed to assess the difference of death and recurrence risks between patient groups with lower and higher number of unfavorable genotype. As shown in Fig. 2, more prominent protective effects conferred by chemotherapy on CRC patients were observed in patient group with higher number of unfavorable genotypes in both overall survival and recurrence-free survival analyses with HRs of 0.28 (95 % CI 0.15-0.52, *P* = 6.02 x 10^-5^) and 0.35 (95 % CI 0.19-0.62, *P* = 3.76 x 10^-4^), respectively (Fig. 2b and d). While less prominent protective effects were observed in patient group with lower number unfavorable genotype in both the overall survival analysis (Fig. 2a) and recurrence-free survival analysis (Fig. 2c).

### Higher order gene-gene interactions and CRC patient clinical outcomes

**Fig. 3:**
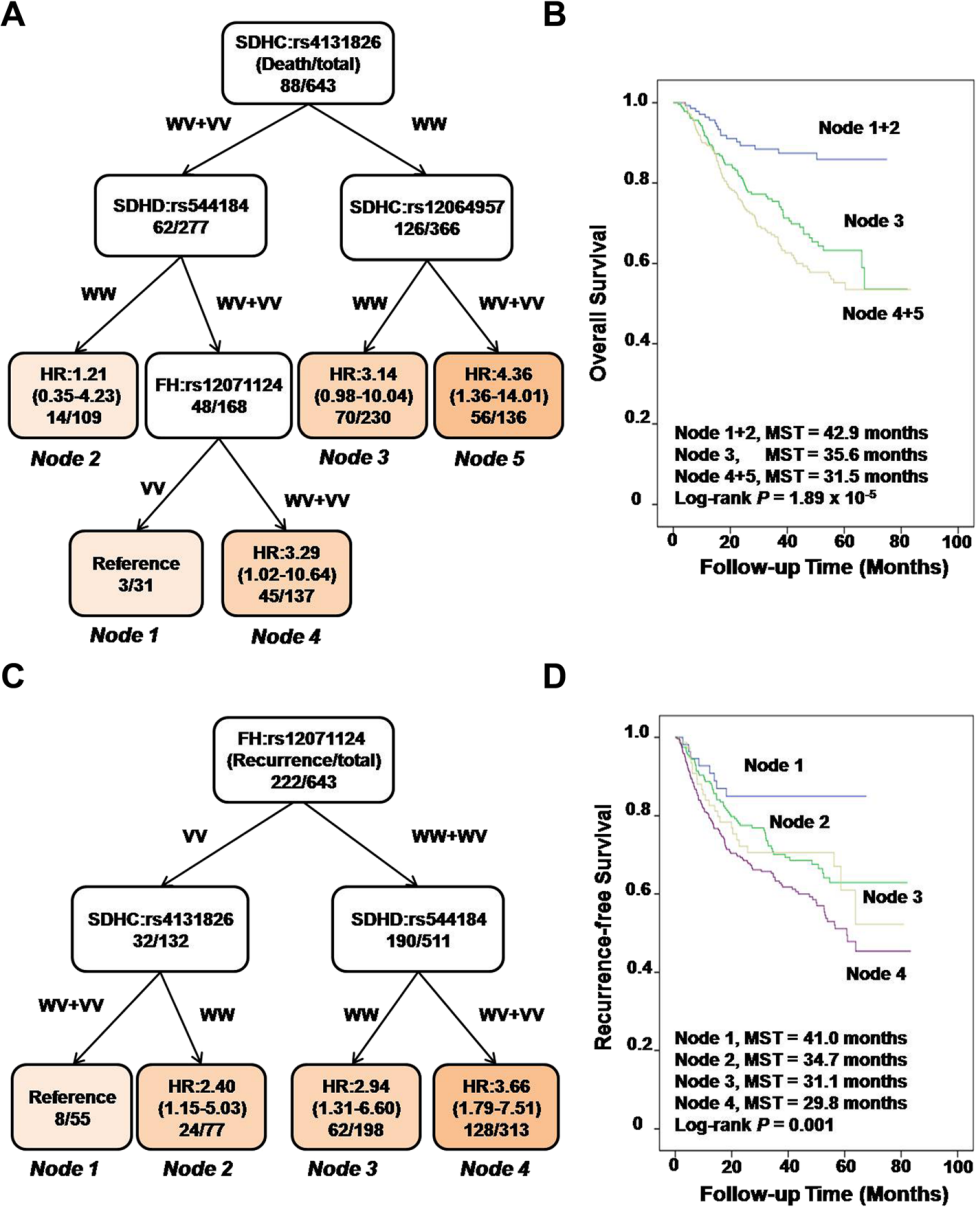
Potential higher order gene-gene interactions among TCA pathway polymorphisms in the overall survival and recurrence-free survival analysis. **a**, **c** Tree structure identifying subgroups of patients with different genetic backgrounds; (**b**, **d**) Kaplan-Meier survival curves for patients based on survival tree analysis

We further explored the higher order gene-gene interactions to reveal whether complex interactions among these significant SNPs could potentially predict the CRC outcomes by survival tree analysis. In the OS analysis, 4 SNPs, including *SDHC*: rs4131826, *SDHC*: rs12064957, *SDHD*: rs544184, and *FH*: rs12071124, exhibited gene-gene interactions, resulting in 5 terminal nodes with different OS times (Fig. 3a). SNP *SDHC*: rs4131826, which initially split the survival trees under dominant model, was the primary factor contributing to the survival difference. Patient groups with lower risk were composed of Node 1 and Node 2 with longest median survival time (MST) of 42.9 months, while patient groups with higher risk were composed of Node 3 with shorter MST of 35.6 months, and Node 4 or Node 5 with shortest MST of 31.5 months (Fig. 3b). In the RFS analysis, another set of 3 SNPs, including *FH*: rs12071124, *SDHC*: rs4131826 and *SDHD*: rs544184, exhibited gene-gene interactions, resulting in 4 terminal nodes with different RFS times (Fig. 3c). The initial split site on the recurrence-free survival tree was due to *FH*: rs12071124, suggesting that this SNP was the primary factor contributing to the recurrence risk difference in our cohort. Longest MST for recurrence was 41.0 months observed in Node1 CRC patients, and significant shorter recurrent MST were observed in Node 2, 3, and 4 patient groups with a *P* value of 0.001 (Fig. 3d).

### Discussion

In the present study, we evaluated the effects of SNPs in genes encoding TCA cycle key metabolic enzymes on the clinical outcomes of 697 CRC patients. We identified 4 SNPs to be significantly associated with CRC death risk and 5 SNPs to be significantly associated with CRC recurrence risk. Cumulative effect analysis showed that these SNPs exhibited significant dose-dependent effects on CRC clinical outcomes. Similar significant results were confirmed in a validation cohort. Further survival tree analysis revealed that SNP rs4131826 in *SDHC* gene and SNP rs12071124 in *FH* gene were the primary factors contributing to OS and RFS, respectively. As the best of our knowledge, our study for the first time comprehensively assessed the association of SNPs in TCA cycle metabolic key enzyme genes and CRC prognosis.

Accumulating evidences have suggested that genetic backgrounds may affect the risk and prognosis of CRC [18–20]. Previous studies have reported that SNPs in genes of most metabolic enzymes, such as glutathione S-transferases, N-acetyltransferase, and cytochrome P450 superfamily of enzymes, can affect enzyme activities and be linked to CRC susceptibility [21–23]. TCA cycle in mitochondria is the core metabolic pathway, which consists of three key metabolic enzymes complexities (SDH, FH and IDH). Recent metabonomic studies have reported that altered metabolite profiles of TCA cycle are observed in serum and urinary samples from CRC patients [24, 25]. Despite the extensive investigations of metabolic enzyme and TCA cycle metabolite markers in CRC, there is no study focusing on the association between SNPs in TCA cycle metabolic enzyme genes and CRC prognosis so far.

In our study, SNP rs4131826 in *SDHC* gene, SNP rs544184 in *SDHD* gene and SNP rs12071124 in *FH* gene were found to be significantly associated with the OS or RFS of CRC patients. IHC analysis further indicated the potential effect of SNPs rs4131826 and rs544184 on the expression of SDHC and SDHD in tissue samples, respectively. Our findings was supported by previous studies showing that the expression of SDHC and SDHD was reduced in tumor tissues and their roles as tumor suppressors have been identified [26–28]. The mutations in genes encoding enzymes of SDH complex and FH in TCA cycle pathway have previously been linked with mitochondrial dysfunction and cancers [11]. Mutations in SDH complex have been initially observed in paraganglioma [29, 30], and subsequent studies have reported those mutations to be involved in T-cell leukemia and gastrointestinal stromal tumor [31, 32]. The potential mechanism of genetic alterations in *SDH* genes associated with cancers has been proposed that these mutations cause the reduction of SDH activity, increase the mitochondrial succinate levels and thus increase mitochondrial reactive oxygen species and oxidative stress [28]. Mutations in FH metabolic enzyme have been observed in multiple uterine leiomyomata and aggressive renal cell carcinoma [33, 34]. FH converts fumarate to malate in the TCA cycle, and *FH* mutations lead to the accumulation of oncometabolite furmarate [35]. Although the specific mechanisms by which SDH/FH is connected with malignancies are probably multifactorial, strong evidences have indicated that dysfunctional cell signaling stimulated by oncometabolites such as succinate and fumurate may be the primary mechanism [6, 8, 13, 36]. In our study, the significant SNPs identified in *SDH* and *FH* genes were all located in the predicted transcriptional factor binding sites (TFBS), suggesting that these SNPs may affect the expression of SDH and FH enzymes. Different enzyme levels may lead to different intracellular concentrations of oncogenic metabolites, resulting in different characteristics of cancer cells which may further affect the prognosis of patients. However further functional studies are needed to reveal the underlying mechanism of our observational results.

Additionally, further stratified analysis indicated that more prominent protective effects conferred by adjuvant chemotherapy after surgical resection on CRC patients were observed in subgroup with higher number of unfavorable genotypes (Fig. 2), in both the overall survival and recurrence-free survival analyses. These results suggest that the SNPs in TCA cycle metabolic enzymes may serve as potential surrogate biomarker for individualized CRC therapy. Although some histological factors such as tumor stage, differentiation and grade, have been identified to be associated with CRC prognosis, there is still a large lack of specific and sensitive biomarker for predicting CRC outcomes [37]. Our findings, together with other molecular biomarker such as *KRAS*, *BRAF* and p53 mutations, may be incorporated into the histological prognostic factors and improve the predictive value in outcomes and treatment response.

Conclusively, our study indicates that genetic variations in TCA cycle metabolic enzyme genes are significantly associated with OS and RFS of CRC patients. Further functional studies are needed to reveal the underlying mechanisms implied in our observational findings.

## Additional files

Additional file 1: Table S1. Distribution of 697 Patients’ Characteristics and Prognosis Analysis. (XLS 25 kb) Additional file 2: Table S2. Distribution of 256 patients’ characteristics and prognosis analysis in validation cohort. (XLS 26 kb) Additional file 3: Table S3. Evaluation of false-positive report probability (FPRP) for SNPs significantly associated with CRC survival. (XLS 26 kb) Additional file 4: Table S4. Cumulative effect of unfavorable genotypes on outcomes of CRC patients in validation cohort. (XLS 20 kb)
